# Evolutionary Diffusion Framework Empowering High-Performance Freeform Terahertz Metasurface Sensing

**DOI:** 10.3390/s26061972

**Published:** 2026-03-21

**Authors:** Chenxi Zhang, Mengya Pan, Qiankai Hong, Shengyuan Shen, Conghui Guo, Yanpeng Shi, Yifei Zhang

**Affiliations:** 1 School of Integrated Circuits, Shandong University, Jinan 250100, China; 2 Shandong Key Laboratory of Metamaterial and Electromagnetic Manipulation Technology, Jinan 250100, China

**Keywords:** terahertz metasurface sensors, deep learning, conditional diffusion model, freeform design, inverse design

## Abstract

Metasurfaces offer an unprecedented avenue to facilitate light-matter interactions. However, traditional design methodologies rely on computationally intensive trial-and-error processes. Moreover, existing deep learning (DL) schemes are predominantly hindered by their massive data requirements and limited exploration of freeform design spaces. To overcome these challenges, a multi-model-driven generative-evolutionary strategy (GES) is proposed, for the on-demand inverse design of bespoke Terahertz (THz) metasurface sensors. By leveraging a Conditional Diffusion Generator (CDG) and an Attention-Enhanced Residual Network (ARN), this framework enables the exploration of an expansive design space encompassing $2^{100}$ possible configurations. The GES effectively overcomes the data bottleneck by selectively generating high-potential data in stages. Full-wave simulations confirm that the inversely designed metasurfaces exhibit high-contrast resonance peaks and exceptional sensitivity across low, mid, and high THz bands. This work provides a versatile paradigm for the efficient design of high-performance functional metamaterials, significantly accelerating the advancement of application-specific THz sensing.

## 1. Introduction

Terahertz (THz) waves refer to electromagnetic (EM) waves that lie between microwaves and infrared waves, within the frequency range of 0.1 to 10 THz [1,2]. Unlike EM waves in other bands (such as X-rays and ultraviolet rays), THz waves are non-ionizing, with photon energy at the meV level, millions of times lower than X-rays, which do not damage the molecular structure of biological samples [3,4]. With the advances in THz wave technology, trace biochemistry substance detection based on THz biological effects has emerged as a promising research direction [5,6,7,8,9]. Although some vibrational modes have been observed in the THz range [10], definitively attributing specific peaks in the THz transmission spectra to the lattice vibrations of target analytes remains challenging [11,12]. Moreover, the relatively long THz wavelength makes it insensitive to small-sized molecules, limiting its application in sensing. To address these challenges, significant attention has been directed toward THz metamaterial sensors. Unlike natural materials with limited spectral responses, metamaterials are artificially engineered periodic structures that derive their properties from the precise design of their geometric shapes, sizes, and spatial arrangements [13]. They are designed to intensely localize EM energy in small volumes, thereby significantly enhancing the interaction of THz waves with matter [14,15,16]. Specifically, metallic resonators patterned on low-loss, high-transparency dielectric substrates have established a reliable paradigm for high-performance THz sensing [17,18,19,20]. However, conventional symmetric or simplistic resonators often struggle to provide the extreme near-field enhancement and multi-modal coupling required for detecting trace analytes. This necessitates the exploration of asymmetric and freeform geometries, which offer far more degrees of freedom to precisely tailor EM resonances and maximize sensitivity.

Realizing application-specific functionalities by tailoring these metallic patterns requires computationally intensive design [21]. Two primary design methodologies, defined as the trial-and-error approach and optimization approach, are prevalently employed in the design of these patterns. In the former, numerical computational methods [22,23], such as finite element method and time-domain finite difference method [24], are commonly utilized to solve Maxwell’s equations on a case-by-case basis. These methods involve parametrically sweeping all possible combinations of geometrical parameters of the metasurface to approximate the desired resonant characteristics. The latter approach employs optimization algorithms, such as topology optimization [25], to manipulate material distributions rather than geometrical structures through filtering and penalization operations. However, these primary methods remain inefficient for customizable metastructure design. For trial-and-error approaches, the computational cost becomes prohibitive as the design degrees of freedom increase. These methods are inherently susceptible to the curse of dimensionality when searching for optimal freeform geometries. Meanwhile, optimization algorithms typically require a complete re-calculation from scratch for every new target resonance. This process lacks the flexibility needed for real-time, on-demand sensing applications. Furthermore, the relationship between complex metasurface patterns and their EM responses is highly non-intuitive. Such complexity makes it difficult for these methods to converge on high-performance designs within a reasonable timeframe. The situation has prompted growing interest in inverse design paradigms that translate target EM responses into metasurface structures [26,27,28,29].

Unlike numerical optimization approaches, deep learning (DL) algorithms leverage learnable data to uncover internal unknown connections, providing a powerful tool to reveal the complex coupling relationship between metasurfaces and incident EM waves [30,31,32,33,34]. Consequently, DL has garnered significant interest in the design of various metasurfaces, such as plasmonic nanostructure [32,35], holographic metasurface [30,31], etc. However, the intrinsic data-driven nature to DL imposes stringent demands on data quality and size, typically exhibiting a non-linear degeneration as the degree of freedom of the metasurface increases [36,37]. This limitation significantly hampers the exploration and application of DL in the global design of metasurfaces. Furthermore, existing works predominantly focus on limited geometrical groups for metasurfaces, such as double openings [38], H-shape [39], cross shape [40], symmetrically cut line shapes [41], etc., which are insufficient to encompass the entire design space with arbitrary geometries. While several recent studies have attempted to expand the design space, they either require the preparation of tens of thousands of datasets in advance [42,43], or encounter intrinsic modeling bottlenecks. Specifically, generative adversarial networks (GANs) [44,45] are limited by mode collapse and training instability [31], which restrict the diversity of the generated structural candidates. Variational autoencoders (VAEs) [46,47] often suffer from reconstruction blurring due to the information loss during latent space compression [31], failing to produce the sharp geometric features required for high-performance sensing. Hence, there is an urgent need to explore advanced generative models beyond traditional GANs and VAEs, along with more efficient training strategies that can maximize data utilization.

In this work, we propose a rapid DL framework for the customized design of planar THz metasurfaces for refractive index sensing applications. We introduce a multi-model-driven generative-evolutionary strategy to achieve the on-demand design of high-performance metasurfaces at high data efficiency. The design and full-wave simulations demonstrate that the purpose-designed metasurface enables the exploration of an almost unlimited design space for the metasurface and shows significant potential for label-free biosensing applications. Our findings establish a new paradigm for the global design of metasurfaces tailored for diverse purposes, which allows for the extension of this approach to other frequency ranges.

## 2. Results and Discussion

### 2.1. Design Paradigm of Freeform Metasurfaces

**Figure 1:**
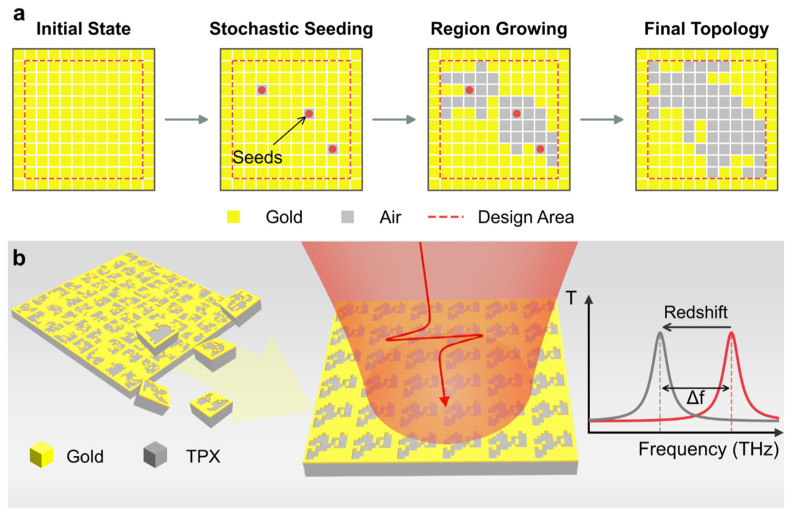
Data preparation. (**a**) Generation process of the freeform meta-atom pattern. (**b**) Schematic of the freeform metasurface that demonstrates a characteristic transmission redshift as the ambient refractive index increases. The structural parameters of metasurface are set as follows: the periodicity is 96 μm; the thickness of the gold layer is set as 100 nm; the thickness of the polymethyl pentene (TPX) substrate is set as 2 mm.

Figure 1 depicts the data acquisition and generation workflow of the freeform metasurface platform, providing the foundational database for the training of subsequent deep learning models. The generation of each data sample involves a two-step process: firstly, a diverse set of meta-atom top-layer metal patterns is created based on a Stochastic Seeded Region Growing (SSRG) strategy; secondly, full-wave Finite-Difference Time-Domain (FDTD) simulations are performed on the generated patterns to obtain and store their corresponding EM responses. The planar metasurface consists of an infinite periodic array of meta-atoms, each following a “metal-dielectric” paradigm. Specifically, the top gold layer in each meta-atom is discretized into a 12 × 12 pixelated grid, with each pixel size as 4 μm × 4 μm. To implement a complementary aperture-type metasurface based on Babinet’s principle, the outermost ring of pixels is fixed as continuous metal. This configuration facilitates the emergence of transmission peaks for sensing [48,49], while ensuring the structural connectivity of the freeform patterns. The inner 10 × 10 designable area offers an astounding $2^{100}$ possible configurations. Specifically, the design complexity of such pixelated metasurfaces scales exponentially as $2^{N}$, where N represents the total number of designable elements. This expansive design space harbors immense potential for generating sophisticated structures that far exceed the scope of conventional trial-and-error methodologies. Their increased degrees of freedom allow for deliberate symmetry breaking and the tailoring of irregular boundaries. Such structural complexity can effectively suppress parasitic resonance modes while maximizing EM field localization within the sensing regions.

The detailed generation process of freeform meta-atom patterns based on the SSRG strategy is illustrated in Figure 1a. Initially, a number of stochastic seeds (typically 2 to 5) are randomly distributed within the 10 × 10 design area. Subsequently, the algorithm enters a recursive region growing phase until the final air-aperture ratio reaches a stochastically sampled value ranging from 0.5 to 0.85. In each iteration, the neighbors of existing air pixels are identified, and a new pixel is randomly selected for activation. This growth mechanism ensures the topological continuity of the dominant air-aperture features while retaining the possibility for a minority of disconnected gold clusters to emerge. By balancing fabrication reliability with structural diversity, this sampling approach efficiently transforms the abstract $2^{100}$ configuration space into a structured dataset of physically realizable meta-atoms.

To facilitate the numerical simulation of metasurfaces, a freeform meta-dataset is created by joint modeling and simulating using Ansys Lumerical FDTD 2023 R2.2 and Python 3.10, respectively. The meta-atoms of THz metasurfaces are set with periodic boundary conditions along the x- and y- directions, and perfectly matched layers (PML) are applied along the z- direction. A y-polarized THz plane wave is employed as the excitation source, propagating along the z- direction. The mesh size is set to 8 μm along the x- and y- directions, and 0.1 μm along the z- direction. The auto-shutoff min threshold is set to $10^{-6}$. The gold conductivity of the upper metal layers is $3.56\times 10^{7}\;S\;m^{-1}$, while TPX substrate is modeled as a lossless dielectric with a refractive index of 1.46. The frequency band of interest ranges from 0.3 to 2.2 THz with 300 sampling points.

**Figure 2:**
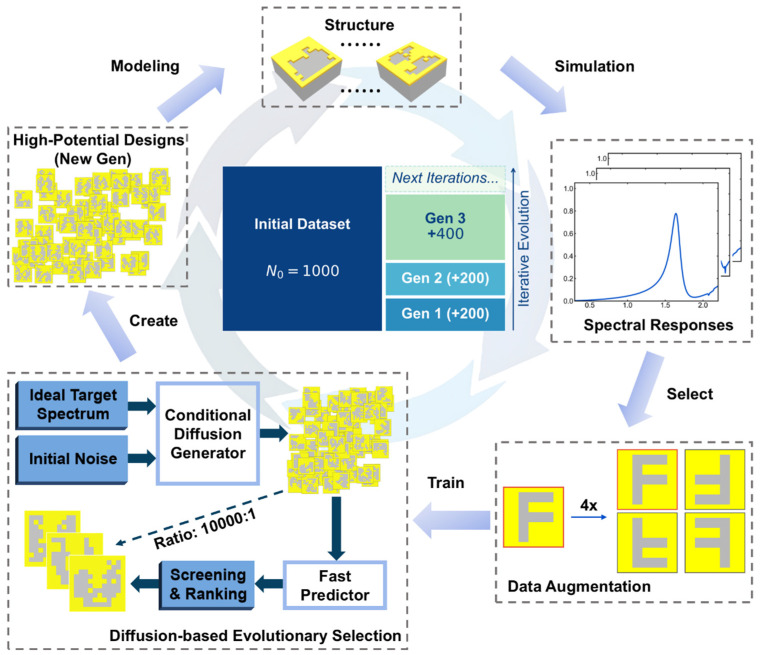
The whole process of generative-evolutionary strategy for metasurface design. It includes five sections: modeling, simulation, selection, training and generation. The central insert represents the iterative evolution of the dataset.

Although the random pixel and SSRG strategy can expand the design space of freeform metasurfaces, the resultant metasurfaces often fail to guarantee superior refractive index sensing performance within the THz frequency range of interest. To address this issue, we propose a Generative-Evolutionary Strategy (GES) for metasurface sensor design, as illustrated in Figure 2, to expand the effective freeform dataset. To facilitate efficient augmentation, we establish a screening criterion prioritizing the Signal-to-Noise Ratio (SNR) before optimizing for sensitivity. For refractive index sensing, a high-quality resonance typically requires a clean background and a high-contrast peak. To quantify this spectral feature, we define a metric termed the Modal Purity Index (MPI). The MPI measures the energy concentration of the main resonance relative to the entire spectrum, defined as

(1) $$\mathrm{MPI}=\frac{\int_{\omega_{c}-\delta }^{\omega_{c}+\delta }{\left|S\left(\omega \right)\right|}^{\;\;\;2}d\omega }{\int_{\omega_{min}}^{\omega_{max}}{\left|S\left(\omega \right)\right|}^{\;\;\;2}d\omega +\epsilon }$$

where $\omega_{c}$ represents the central frequency of the dominant peak, δ denotes the half-window width (set to 10 frequency points), and ϵ is a small constant to prevent division by zero. A higher MPI indicates a sharper resonance with a clean background. Subsequently, under the proposed GES framework, the initial phase (Gen 1–3) prioritized the MPI and peak height, while the sensitivity metric was incorporated starting from Gen 4. The complete implementation of the GES flowchart is shown below.

First, following the joint simulation scheme, 1000 datasets of freeform metasurfaces are generated and simulated as the initial generation of input dataset. To facilitate efficient data utilization, data augmentation techniques (Supplementary Note S2 and Figure S1, Supporting Information for details) are employed on the patterns before training the DL models. Notably, we employ a differentiated training strategy for the two models. The forward predictor is trained on the entire dataset to ensure a comprehensive understanding of the global design space and robust generalization. In contrast, the diffusion-based generator is trained exclusively on screened high-quality samples. This is because diffusion models intrinsically learn to approximate the underlying data distribution; including low-performance samples would dilute the feature space, causing the model to generate suboptimal designs.

Initially (Gen 1–2), the generator was conditioned on the spectra of screened high-quality samples to facilitate rapid local refinement. However, relying solely on historical data limits the exploration to the existing distribution. To break this bottleneck, we construct a library of idealized Lorentzian target spectra as conditional inputs for the generator starting from Gen 3. Here, 100 distinct target spectra are predefined by the idealized Lorentzian profile, with resonant centers ($f_{c}$) uniformly sampled across 0.4–2.1 THz, expressed as

(2) $$T\left(f\right)=\frac{1}{1+{\left(\frac{f-f_{c}}{\gamma /2}\right)}^{2}}$$

where γ represents the target bandwidth (set to 0.2 THz). For each $f_{c}$, the conditional diffusion generator produces a vast pool of candidates, which are filtered by the fast predictor with a 10,000:1 screening ratio. To implement this, we first apply thresholds on peak height to eliminate unqualified designs. Subsequently, the remaining candidates are ranked by their MPI, and the top-ranked designs are selected. These high-potential designs are then validated via the joint simulation scheme and merged into the training pool to update the dataset for the next iterative cycle.

**Figure 3:**
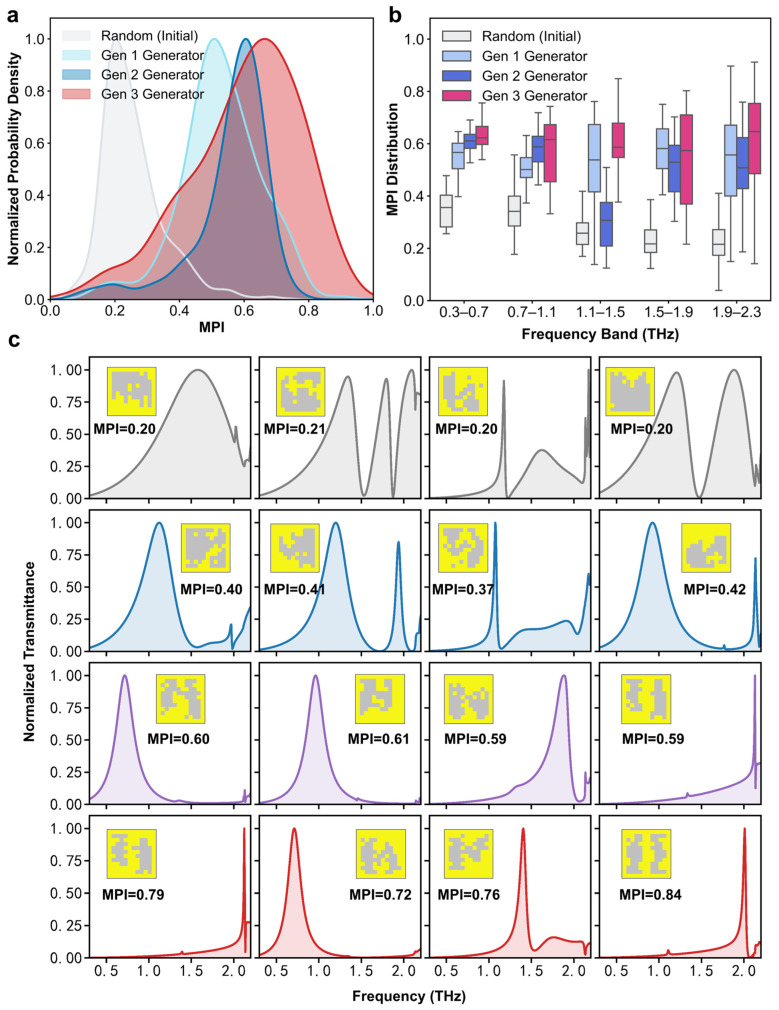
Evolution of MPI in the generated metasurfaces. (**a**) Probability density distribution of MPI. (**b**) Frequency-dependent MPI distribution. (**c**) Representative spectra of varying MPIs.

From the distribution of the MPI response in each generation shown in Figure 3a, the number of metasurfaces with improved MPI increases steadily with each incremental iteration cycle. Notably, the distribution for Gen 3 exhibits a distinct broadening compared to the previous generations. Unlike the localized refinement in Gen 1–2, the introduction of idealized Lorentzian targets (Equation (2)) compels the generator to explore a much vaster and more diverse structural space. While it successfully pushes a significant proportion of designs into the high-MPI region, it also inherently entails a broader spread that includes necessary low-MPI trials. Specifically, the inclusion of these lower-MPI samples provides the predictor with essential negative feedback, allowing it to more accurately identify and filter out low-MPI candidates in subsequent generations. By iteratively incorporating these mispredicted samples into the training pool, the predictor progressively matures, thereby refining the overall efficiency of the evolutionary cycle. Furthermore, Gen 2 showed an overall average MPI improvement compared to Gen 1, but experienced degradation in multiple frequency bands, as shown in Figure 3b. Gen 3 achieves a higher proportion of high-MPI designs across all frequency bands compared to previous generations, demonstrating that the proposed GES method offers an effective evolutionary strategy for generating numerous metasurfaces characterized by a high degree of freedom.

As shown in Figure 3c, at low MPI levels (around 0.2), spectra exhibit either overwhelming resonance broadening or severe multi-peak interference. As the MPI increases to approximately 0.4, parasitic modes subside and the primary peak narrows, though some dual-peak features persist. Upon reaching high MPI levels (around 0.6), dual-peak interference completely disappears, resulting in a clean, narrow single resonance that is favorable for peak detection. As the MPI further increases beyond 0.7, the EM energy becomes more concentrated, yielding exceptionally sharp resonance peaks that significantly enhance the sensing resolution. Given these distinct spectral signatures, one would expect the screening process to eliminate all low-quality candidates. However, despite the stringent 10,000:1 screening ratio, a small fraction of validated samples still falls into the low-MPI region. Because the fast predictor struggles to achieve absolute precision under limited data conditions, occasionally overestimating the MPI of certain samples. In deep learning-aided design, data scarcity is a prevalent challenge. Crucially, even with an imperfect predictor, the GES framework remains highly effective in improving design metrics. By iteratively incorporating these mispredicted samples back into the training pool, the predictor also progressively matures through the evolutionary cycle.

### 2.2. Global Design Model Construction

Our global design framework acts as a dynamic engine composed of a Fast Predictor and a Conditional Diffusion Generator, which are iteratively updated within each cycle of the GES. To evaluate the model performance, the dataset is divided into the training, validation and test sets, with proportions of 80%, 10% and 10%, respectively.

#### 2.2.1. Fast Prediction Network

**Figure 4:**
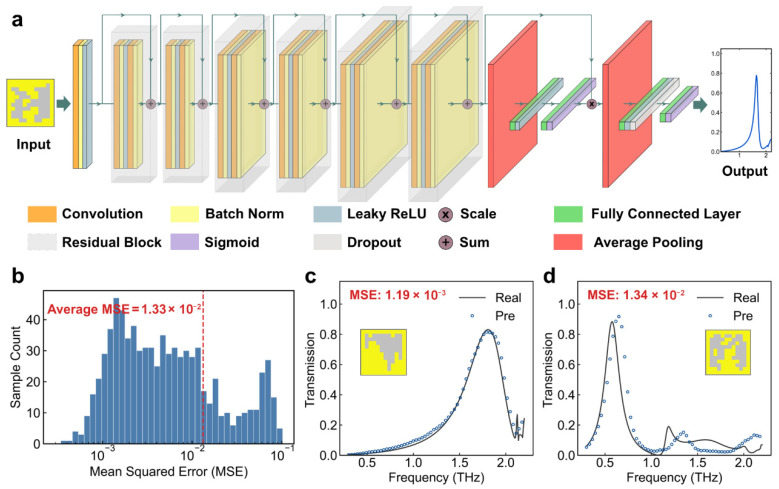
Architecture and Performance of the Attention-enhanced Residual Network (ARN). (**a**) ARN based on the deep residual network ResNet18 (architecture shown for spectral response prediction as an example). (**b**) Statistical distribution of the MSE for the spectral prediction task on the test set. (**c**,**d**) Representative spectral prediction results for samples with different MSE levels.

A pre-trained forward prediction network is necessary for predicting and screening high-potential designs. This prediction task is executed by two specialized networks: the first network is trained to predict spectral responses for screening the MPI and peak height throughout the entire GES process, while the second network is trained to estimate sensor sensitivity to further refine candidates in Gen 4. To address these demands, an attention-enhanced residual network (ARN) based on the residual network ResNet18 [50] is proposed, as shown Figure 4a. Two specialized networks use the ARN architecture, with the only variation being the output dimension of the final fully connected layer.

The choice of ResNet18 is motivated by its ability to effectively extract deep features from freeform patterns. Specifically, our implementation employs a progressive channel scaling strategy, where feature dimensions transition from 32 to 128 across six sequential residual layers. This tiered design enables the network to extract multi-scale geometric features, ranging from local pixel correlations to global topological symmetries. Furthermore, the ARN incorporates a self-attention mechanism. Feature maps from the final residual block are compressed via Average Pooling and processed through a sequence of feature extraction layers to generate channel-wise weights. These weights are subsequently multiplied back onto the original feature maps via a Scale operation, thereby prioritizing EM-sensitive features (See Supplementary Note S1 and Table S1, Supporting Information for more details about the ARN).

For spectral response prediction, the objective is to screen the MPI and peak height, which are features inherently dependent on the global spectrum. A standard Mean Squared Error (MSE) loss is employed to maintain high prediction accuracy across the entire frequency range:

(3) $$L_{MSE}=\frac{1}{N}\sum_{i=1}^{N}{\left(y_{pred,i}-y_{true,i}\right)}^{2}$$

In contrast, for sensitivity estimation, the primary concern is the reliability of the screening process. Given our 10,000:1 screening mechanism, preventing the overestimation of sensitivity is more critical than avoiding underestimation. Furthermore, calculating sensor sensitivity typically requires simulations under varying ambient indices (*n*), which is computationally expensive and results in more limited training data. To address this issue, we propose an Asymmetric Screening Loss (ASL) based on the Huber loss, incorporating a non-symmetric dynamic weighting strategy:

(4) $$L_{ASL}=\frac{1}{N}\sum_{i=1}^{N}\left[L_{H}\cdot \left(1+M\cdot \left(P_{base}+\eta {\left(y_{pred}-y_{true}\right)}^{2}\right)\right)\right]$$

where $L_{H}$ epresents the Huber loss (with δ=0.3), *M* is a binary mask activated for overestimations ($y_{pred}>y_{true}$), and η=10 is the penalty scaling factor for the quadratic error term.

The predictive efficacy of the trained Gen-4 ARN is evaluated on an independent test set of 800 samples. Figure 4b–d demonstrate the ARN’s effectiveness by showcasing samples from the test dataset, including corresponding patterns and prediction errors. While discrepancies occur in a small subset of samples with larger MSEs, the majority of predictions are highly accurate, meeting the screening requirements within the GES framework (Supplementary Note S3 and Figure S2, Supporting Information for more samples).

#### 2.2.2. Inverse Design Network

**Figure 5:**
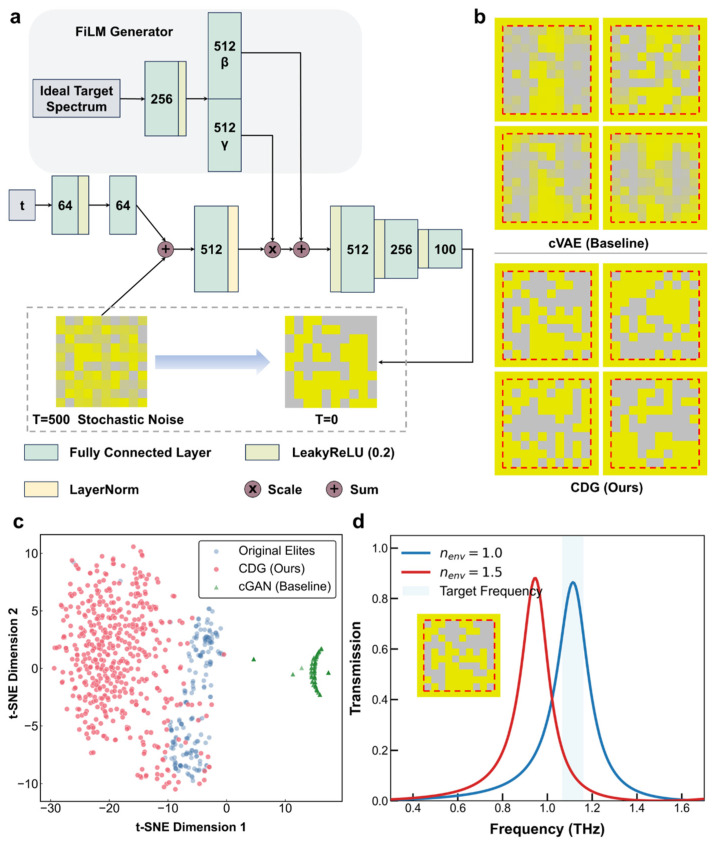
Architecture and Performance of the Conditional Diffusion Generator (CDG). (**a**) Architecture of the CDG based on the denoising diffusion probabilistic model (DDPM). (**b**) Comparison of geometric fidelity between the cVAE and the CDG. (**c**) Comparison of structural diversity between the cGAN and the CDG. (**d**) Representative inverse design instance.

Within the proposed GES framework, the generator produces a vast pool of patterners candidates with higher potential than the SSRG strategy. This efficient generation capability is fundamental to the subsequent 10,000:1 screening process. To ensure this stringent selection does not converge on limited patterns, maintaining the diversity of these high-potential patterns is essential for a comprehensive exploration of the design space. To address these demands, a Conditional Diffusion Generator (CDG) based on the Denoising Diffusion Probabilistic Model (DDPM) [51] is proposed, as shown in Figure 5a. This network focuses on ensuring the final filtered results meet the requirements, rather than aiming for the precision of any single generation.

The choice of the DDPM framework is primarily motivated by its superior ability to preserve structural diversity while maintaining high geometric fidelity. Unlike GANs that are prone to mode collapse [31] and VAEs that often suffer from reconstruction blurring [31], the diffusion framework offers a more stable and diverse generative performance. Unlike a standard DDPM, where samples are denoised from a standard normal distribution to generate synthetic patterns resembling the training data, the CDG introduces a conditional injection mechanism. As illustrated by the gray shaded area in Figure 5a, this mechanism is realized through the Feature-Wise Linear Modulation (FiLM). The FiLM Generator maps the ideal target spectrum into two sets of modulation vectors, γ and β. To enhance numerical stability during the denoising process, a damping effect is integrated into the affine transformation:

(5) $$h^{\prime }=\left(1+0.1\cdot \tanh\left(\gamma \right)\right)⊙h+\left(0.1\cdot \beta \right)$$

where *h* represents the intermediate feature maps of the backbone and ⊙ denotes element-wise multiplication. By treating the inverse design as a conditional denoising process, the CDG can map initial stochastic noise (*T* = 500) to a diverse array of sharp patterns (*T* = 0) based on idealized spectral requirements.

To verify the effectiveness of the CDG, we conducted a comparative analysis with the cVAE and cGAN baselines. For a fair comparison, all three models were implemented with equivalent network capacities and trained using the same dataset derived from the Gen 3 elite pool. The idealized Lorentzian spectra defined in Equation (2) served as the conditional input. The CDG achieves significantly higher geometric fidelity than the cVAE, as shown in Figure 5b. While the cVAE outputs are characterized by blurred boundaries, the CDG produces sharp patterns with unambiguous binarized topologies. To quantitatively evaluate this, we calculated the Mean Binarization Deviation (MBD), defined as

(6) $$MBD=\frac{1}{N}\sum \min\left(p,1-p\right)$$

where p represents the pixel value. A lower MBD indicates higher geometric contrast and better binarization. Our analysis shows that the CDG achieves a near-ideal MBD of approximately 0.0001, while the cVAE baseline exhibits a much higher deviation of 0.0895. This over 800-fold improvement in MBD confirms that the CDG effectively eliminates the reconstruction blurring common in VAE-based architectures. Furthermore, the CDG successfully avoids the mode collapse inherent in the cGAN, as shown in the t-SNE visualization in Figure 5c. This confirms that the CDG possesses a superior capability to explore the design space, providing the necessary structural diversity for identifying global optima.

Figure 5d illustrates a closed-loop instance of the inverse design process. Upon a user-defined target frequency, which serves as the $f_{c}$ in Equation (2) to construct the conditional input. Guided by this condition, the generator produces a vast pool of candidates that are then filtered by the fast predictor. Full-wave verification confirms that the resulting design aligns with the target. Moreover, the spectrum exhibits a distinct redshift when the ambient refractive index increases, demonstrating the sensing effectiveness of the inversely designed metasurface.

### 2.3. Characterization of Designed Metasurface

**Figure 6:**
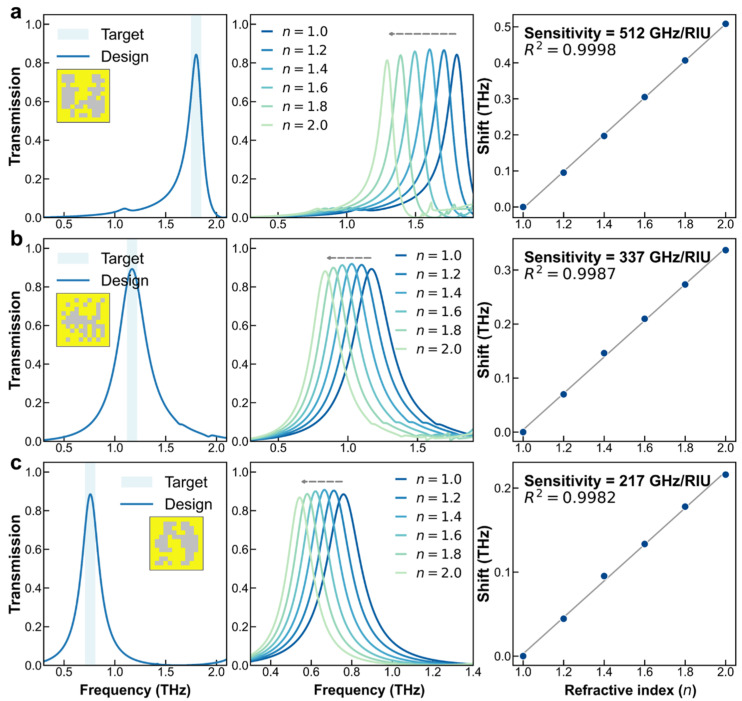
Sensing performance analysis of the inversely designed metasurfaces. (**a**–**c**) Representative inverse design examples for high, mid, and low frequencies, respectively. Left: Inversely designed patterns and their full-wave verification based on user-defined targets. Middle and Right: Spectral evolution and linear sensitivity fitting with different refractive index.

To further validate the sensing capability of the customized metasurfaces, inverse design instances were performed across various frequency regimes, as shown in Figure 6. Guided by the proposed GES, the screening process accounts for peak height, spectral background purity, and sensing sensitivity. Specifically, the user-defined resonance frequency serves as the primary target. Subsequently a multi-criteria scoring strategy is employed to evaluate the candidates generated in the inverse design stage. Potential designs are required to satisfy stringent physical thresholds, such as a peak intensity greater than 0.6 and a sensitivity exceeding 0.2 THz/RIU. The remaining candidates are then ranked by a composite score that balances the MPI with frequency accuracy. This modular screening approach also allows for the seamless integration of additional constraints, including minimum MPI requirements and fabrication-related geometric limits. Consequently, the inversely designed metasurfaces exhibit high-contrast resonance peaks with minimal parasitic modes. This superior spectral quality is consistent across the high, mid, and low THz bands. Furthermore, these freeform structures maintain exceptional linearity and high sensitivity throughout the frequency range of interest. These results confirm the robustness and precision of the design framework for high-performance sensing applications.

## 3. Conclusions

We present an advanced DL framework tailored for the customized design and optimization of bespoke THz metasurface sensors. Our proposed design strategy leverages staged generation of high-potential data tailored to target requirements, significantly reducing both the data collection scale and computational demands. Unlike traditional methodologies that heavily rely on simulations to obtain comprehensive data, our approach efficiently captures the most beneficial subsets of the comprehensive design space while maintaining high exploration freedom. By integrating the CDG inverse design model with ARN into a comprehensive global design framework, we effectively filter out low-performance metasurfaces. This ensures that the EM response adheres to the specified design criteria, enabling on-demand design of customized metasurface sensors to enhance sensing capabilities. Our efforts have resulted in the generation of high-quality datasets, an expansion of the design space for metasurfaces, a solution to the data bottleneck in DL, and a significant improvement over traditional design methods. Notably, our framework exhibits exceptional fault tolerance. It does not require meticulous model design or high accuracy in data-scarce regimes, as the evolutionary cycle allows the system to progressively mature through iterative feedback. This framework can be adapted to various sensing requirements by simply modifying the target metrics. Furthermore, the proposed GES framework is fundamentally material-agnostic and can be readily extended to purely dielectric metasurfaces by retraining the models with high-index dielectric datasets. Beyond planar topologies, the framework’s flexibility also allows for expansion into 3D structural designs by incorporating height as an additional design degree of freedom. This provides a versatile paradigm for the efficient design of high-performance metamaterial sensors, thereby accelerating the realization of next-generation functional devices.

## Data Availability

The raw data supporting the conclusions of this article will be made available by the authors on request.

## Supplementary Materials

The following supporting information can be downloaded at: https://www.mdpi.com/article/10.3390/s26061972/s1, Supplementary Note S1: ARN Model Architectures; Supplementary Note S2: Data Augmentation; Supplementary Note S3: The predictive performance of ARN; Table S1: Architectural specifications of the ARN; Figure S1: Data augmentation methods; Figure S2: Predictive performance of the ARN.

## Abbreviations

The following abbreviations are used in this manuscript:

DL	Deep learning
THz	Terahertz
EM	Electromagnetic
GES	Generative-evolutionary strategy
SSRG	Stochastic Seeded Region Growing
CDG	Conditional Diffusion Generator
ARN	Attention-enhanced Residual Network
GANs	Generative adversarial networks
VAEs	Variational autoencoders
DDPM	Denoising diffusion probabilistic model
TPX	Polymethyl pentene
FDTD	Finite-Difference Time-Domain
PML	Perfectly matched layers
MPI	Modal Purity Index
SNR	Signal-to-Noise Ratio
MSE	Mean Squared Error
ASL	Asymmetric Screening Loss
MBD	Mean Binarization Deviation
FiLM	Feature-wise Linear Modulation
