# Supplementary material for: Evolutionary Diffusion Framework Empowering High-Performance Freeform Terahertz Metasurface Sensing
Source: Sensors (Basel). 2026 Mar 21;26(6):1972. doi: 10.3390/s26061972 (PMC13029974; doi:10.3390/s26061972)
Supplement: Supplementary file 1 [file sensors-26-01972-s001.zip › sensors-4192598-supplementary.pdf]

Supplementary Information for

Evolutionary diffusion framework empowering high-performance freeform terahertz metasurface sensing

Chenxi Zhang[1], Mengya Pan[1,2], Qiankai Hong[1], Shengyuan Shen[1], Conghui Guo[1], Yanpeng Shi[1,2], Yifei Zhang[1,2]

1 School of Integrated Circuits, Shandong University, Jinan 250100, China;

2 Shandong Key Laboratory of Metamaterial and Electromagnetic Manipulation Technology, Jinan 250100, China

(* Yanpeng Shi and Yifei Zhang are co-corresponding authors: ypshi@sdu.edu.cn, yifeizhang@sdu.edu.cn)

The PDF file includes:

Supplementary Note S1: ARN Model Architectures...2

Supplementary Note S2: Data Augmentation...2

Supplementary Note S3: The predictive performance of ARN..............................3

Supplementary Note S1: ARN Model Architectures.

The architecture of the Attention-Enhanced Residual Network (ARN) is shown in Table S1. Specifically, the output dimension of the final fully connected layer (FC 4) is set to 300 for spectral response prediction, whereas it is set to 1 for sensitivity prediction.

Table S1. Architectural specifications of the ARN.

Layer	Input	Output	Kernel
Initial Convolution	1×10×10	32×10×10	32×3×3
Residual Block 1-2	32×10×10	32×10×10	32×3×3, identity
Residual Block 3	32×10×10	64×10×10	64×3×3, 64×1×1
Residual Block 4	64×10×10	64×10×10	64×3×3, identity
Residual Block 5	64×10×10	128×10×10	128×3×3, 128×1×1
Residual Block 6	128×10×10	128×10×10	128×3×3, identity
Average Pooling	128×10×10	128×1×1	-
FC 1 (Squeeze)	128	16	-
FC 2 (Excitation)	16	128	-
FC 3	128	512	-
FC 4 (Output)	512	300	-

Supplementary Note S2: Data Augmentation.

To enhance the generalization and mitigate overfitting of the deep learning models, a four-fold data augmentation strategy is implemented. Each pixelated pattern undergoes 180° rotation, vertical flipping, and horizontal flipping, expanding the dataset size by a factor of four. The physical validity of this strategy is verified using an asymmetric F-shaped aperture pattern, as shown in Figure S1. The simulated transmission spectra demonstrate perfect consistency across all four variations. From the perspective of the neural network, although the spectra responses remain consistent, the input pixel

matrices representing these rotated or flipped patterns are transformed into entirely different feature distributions. By introducing these diverse distributions, the model effectively learns underlying geometric symmetries and decouples physical signatures from absolute spatial coordinates. This process significantly enhances the model's robustness and generalization capability, which is particularly vital in data-scarce regimes. Consequently, the deep learning models can reliably utilize these augmented samples to learn structural diversity without introducing physical bias.

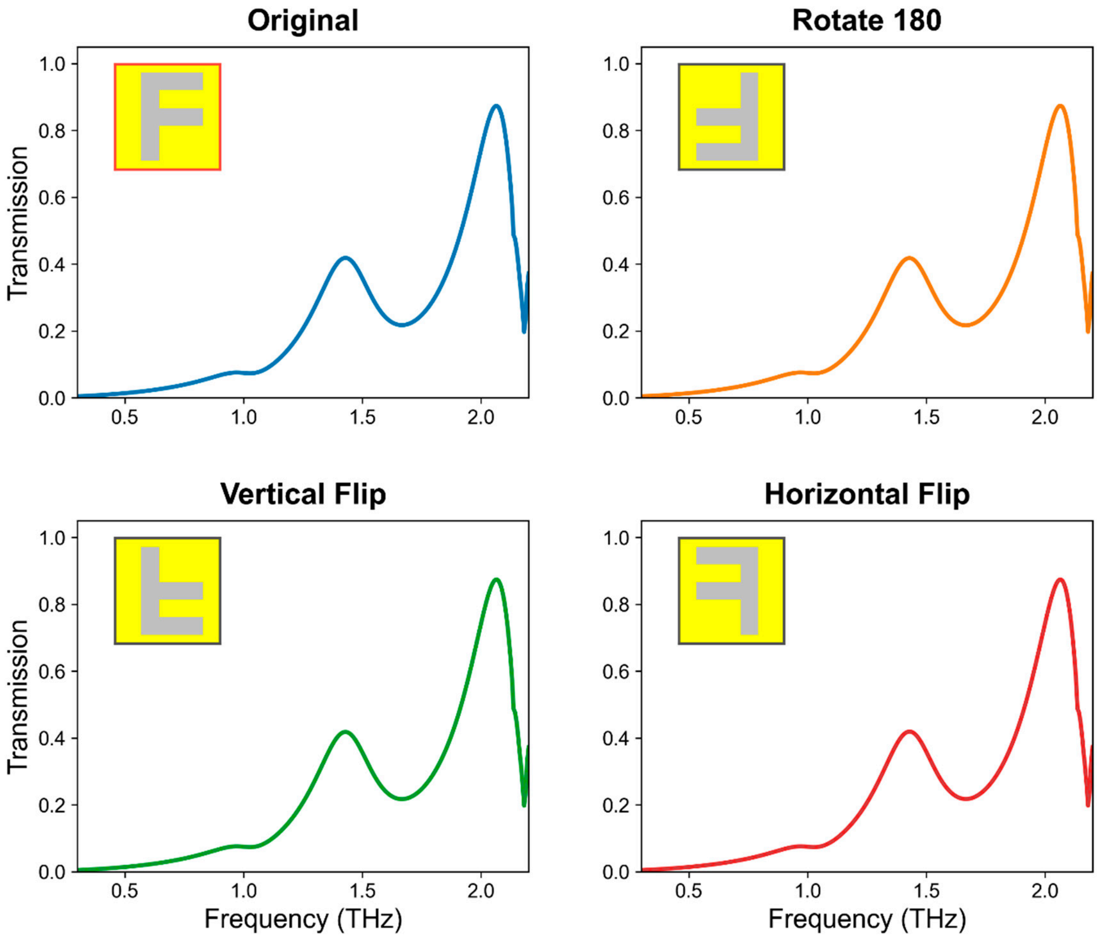

Figure S1. Data augmentation methods.

Supplementary Note S3: The predictive performance of ARN.

To further demonstrate the predictive capability of the Attention-Enhanced Residual

Network (ARN), we provide additional representative instances of spectral response predictions on the independent test set in Figure S2. A robust consistency exists between the MSE metric and the prediction accuracy of the spectral response. When the MSE is low, the predicted spectral responses show near-perfect overlap with the full-wave simulation results. Consequently, this high-fidelity reconstruction ensures precise estimation of the Modal Purity Index (MPI) and peak height. As the MSE increases, although the predicted curves may no longer perfectly overlap with the simulation results, the overall spectral trends and primary resonance positions remain correctly identified. Even with minor localized deviations, the network maintains its ability to provide an approximation of the MPI and peak height. This confirms that the ARN functions as an effective screening tool within the GES framework. Despite prediction inaccuracies, it successfully filters out low-quality designs and retains high-potential candidates for subsequent evolutionary cycles.

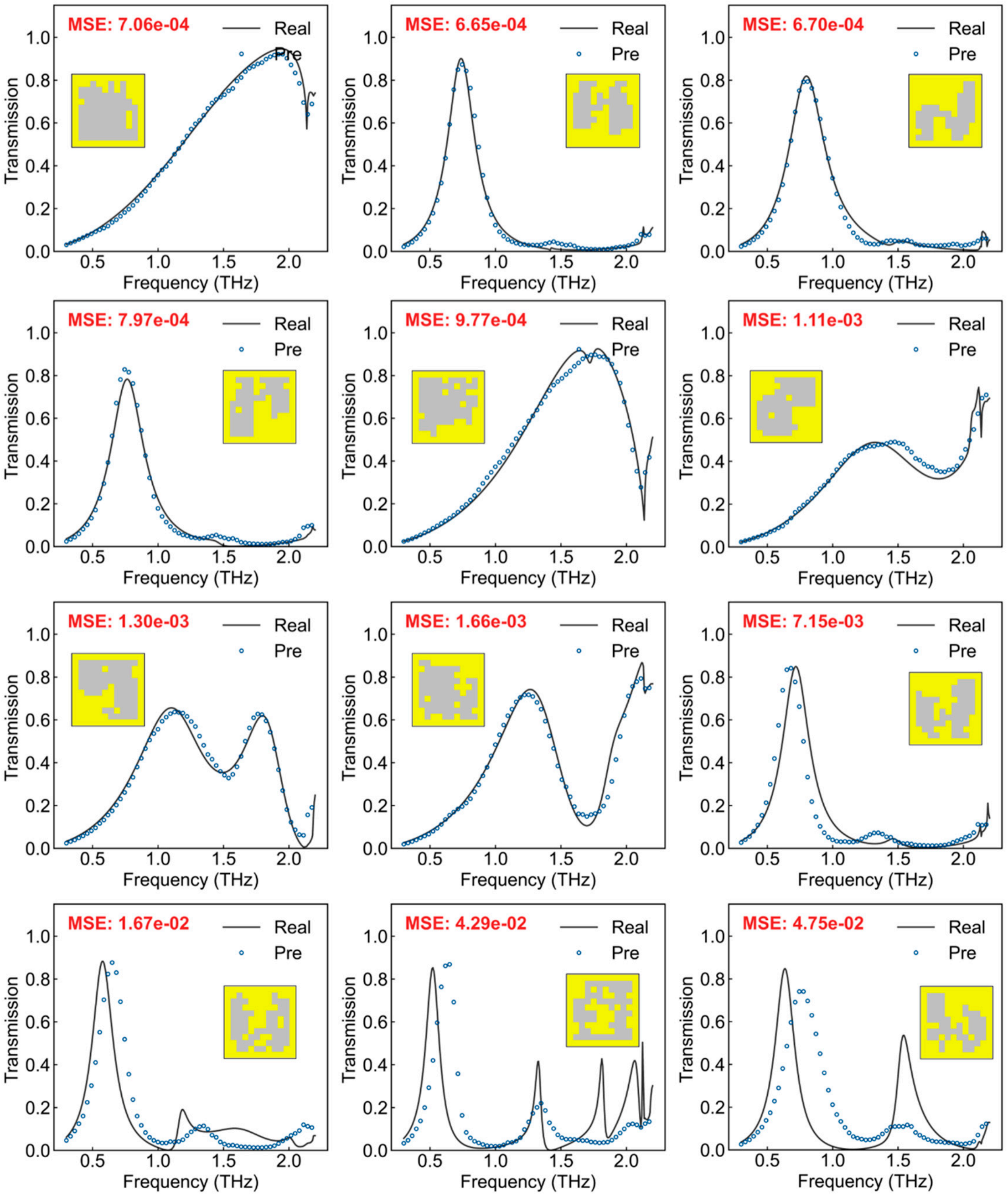

Figure S2. Predictive performance of the ARN.
